# Plasma metabolomics reveals major changes in carbohydrate, lipid, and protein metabolism of abruptly weaned beef calves

**DOI:** 10.1038/s41598-023-35383-2

**Published:** 2023-05-20

**Authors:** Luciano A. González, Julia G. S. Carvalho, Bruno C. Kuinchtner, Anthony C. Dona, Pietro S. Baruselli, Michael J. D’Occhio

**Affiliations:** 1 Sydney Institute of Agriculture, and School of Life and Environmental Sciences, Faculty of Science, The University of Sydney, Camden, NSW 2570 Australia; 2 Departamento de Reprodução Animal, Faculdade de Medicina Veterinária e Zootecnia, Universidade de São Paulo, São Paulo, SP Brazil; 3 Natural Pasture Ecology Laboratory (LEPAN), Universidade Federal de Santa Maria, Santa Maria, RS Brazil; 4 Kolling Institute of Medical Research, Northern Medical School, University of Sydney, St Leonards, NSW 2065 Australia

**Keywords:** Animal behaviour, Animal physiology

## Abstract

^1^H NMR-based metabolomics was used to study the effect of abrupt weaning on the blood metabolome of beef calves. Twenty Angus calves (258 ± 5 kg BW; 5 to 6 months old) were randomly assigned to a non-weaned (NW) group that remained grazing with their dam or a weaned (W) group that underwent abrupt separation from their dam to a separate paddock on d 0 of the study. Body weight, behaviour, and blood samples for cortisol and metabolomics were measured at d 0, 1, 2, 7, and 14 of the study. On d 1 and 2, W calves spent less time grazing and ruminating, and more time vocalising and walking, had a greater concentration of cortisol, NEFA, 3-hydroxybutyrate, betaine, creatine, and phenylalanine, and lesser abundance of tyrosine (*P* < 0.05) compared to NW calves. Compared to NW calves at d 14, W calves had greater (*P* < 0.01) relative abundance of acetate, glucose, allantoin, creatinine, creatine, creatine phosphate, glutamate, 3-hydroxybutyrate, 3-hydroxyisobutyrate, and seven AA (alanine, glutamate, leucine, lysine, phenylalanine, threonine and valine) but lesser (*P* < 0.05) relative abundance of low density and very low-density lipids, and unsaturated lipids. Both PCA and OPLS-DA showed no clustering or discrimination between groups at d 0 and increasing divergence to d 14. Blood metabolomics is a useful tool to quantify the acute effects of stress in calves during the first 2 days after abrupt weaning, and longer-term changes in carbohydrate, lipid and protein metabolism due to nutritional changes from cessation of milk intake and greater reliance on forage intake.

## Introduction

Nutritional requirements of new-born and pre-ruminant beef calves are met exclusively from protein, fat, carbohydrates (mainly lactose), vitamins and minerals absorbed from milk that bypasses the undeveloped rumen^1^. The transition from the pre-ruminant to the ruminant stage in beef calves raised on pasture occurs gradually as consumption of solid feed and rumen fermentation increases and the animal becomes more reliant on microbial protein and VFA^2,3^. One of the most outstanding features of the ruminant animal is the high gluconeogenic potential from propionate, lactate and AA^1^. The abrupt removal of a calf from its dam (weaning) at 3 to 6 months of age is a standard practice in beef cattle production systems^4^. Weaned calves may experience nutritional, social, and psychological stress which causes behavioural, metabolic, physiological, and immunological changes^5,6^. Therefore, weaning practices should consider animal welfare, metabolic function, and nutritional requirements of the calves^2^. Previous studies have focussed on changes in behaviour, stress hormones, and immunology of weaned calves, which are more evident during the first 48 h after weaning^5,7–9^. The catabolic effect of stress hormones such as cortisol can explain the increased concentrations of NEFA in blood and some of the decline in ADG after weaning^10,11^. However, the cessation of milk intake of calves at pasture is expected to produce longer-term metabolic changes because dietary fat is reduced due to the low concentration in forages whereas fiber and ruminal fermentation increases. Surprisingly, little is known about changes in metabolic function of grazing calves after weaning. This information could be used to develop new strategies to alleviate metabolic stress, improve welfare, and minimize the decline in animal production after weaning.

Metabolic function is reflected in the blood metabolome which comprises metabolites such as lipids, sugars and AA that influence cellular, tissue and organ function^12,13^. The metabolome is downstream of the genome, transcriptome, and proteome, and is the closest ‘omic’ to the phenotype. This led to the suggestion that the metabolome may be the best indicator of alterations in biological function^14–16^. Another important feature of the blood metabolome is that it represents the integration of external (e.g., diet) and internal (e.g. genotype) factors that influence metabolism^15^. In pigs, weaning was associated with changes in both metabolic function and the blood metabolome and these changes could be partially countered by supplementation with arginine^17^. A previous study reported that neonatal calves fed colostrum showed an increase in serum abundance of glutamate, histidine, methionine, phenylalanine, tyrosine, tryptophan, valine, leucine, isoleucine and proline, and a reduction of glutamine^13^. However, no studies were published assessing changes in the blood metabolome of grazing beef calves after weaning under commercial conditions.

The aim of the present study was to characterize changes in the blood metabolome of Angus beef calves subjected to abrupt weaning. This information could improve our understanding of the metabolic impact of abrupt weaning in beef calves and inform novel strategies to manage the transition. The hypothesis was that abrupt weaning in beef calves was associated with stress and nutritional changes that produce metabolic responses reflected in the blood metabolome.

## Materials and methods

### Animal ethics

The study had animal ethics approval from The University of Sydney Animal Ethics Committee: Protocol 767. All methods were performed in accordance with ethics approval and the relevant State and National acts, regulations and codes including Animal Research Act 1985 (https://www.dpi.nsw.gov.au/about-us/legislation/list/animal-research), Animal Research Regulation 2021 (https://legislation.nsw.gov.au/view/pdf/asmade/sl-2021-477) and Australian code for the care and use of animals for scientific purposes 8th Edition 2013 (https://www.nhmrc.gov.au/about-us/publications/australian-code-care-and-use-animals-scientific-purposes).

### Animals and management

Twenty multiparous Angus cows and their calves (5 to 6 months old) were maintained under standard grazing conditions and management at The University of Sydney John Pye Farm (Greendale, New South Wales, Australia). The pastures were dominated by Paspalum (*Paspalum dilatatum*) with relatively low densities of Chicory (*Cichorium intybus*), Plantain (*Plantago lanceolata*), Red Clover (*Trifolium pratense*) and White Clover (*Trifolium repens*) and contained 10.7% CP, 60.7% NDF, and 20.6% ADF. Calves were spring born and had remained with their dam from birth.

### Study design and sampling

On d 0, calves were weighed, blocked by sex, and randomly assigned to a non-weaned group that remained with their dam (NW; *n* = 10; 257 ± 6 kg BW) or a weaned group that underwent abrupt separation from their dam (W; *n* = 10; 258 ± 5 kg BW) and sent to a separate paddock with no visual or auditory contact with their dam. Each group had 7 male (non-castrated) and 3 female calves. Pasture in the two paddocks was similar in composition and quantity. This was verified by pasture sampling and analysis at the start (d 0) and end (d 14) of the study. For this purpose, ten quadrats (0.5 m × 0.5 m) were randomly distributed in each paddock and the forage inside the quadrats was cut at ground level, dried for 48 h at 65 °C, and weighed to calculate available forage biomass. The two groups of calves remained in their respective paddock from d 0 to end (d 14) of the study. Body weight was recorded for all calves at d 0, 2, 7 and 14. Blood samples were collected by jugular venipuncture using evacuated tubes containing EDTA (BD Vacutainer, Becton Dickinson, NJ, USA) at d 0, 1, 2, 7, and 14 between 09:00 AM and 10:00 AM. Samples were refrigerated at 4 °C for approximately 30 min, centrifuged (2000×*g* for 30 min), and plasma stored at − 80 °C until metabolomics analysis.

Behaviour was measured using scan sampling at 3 min intervals for each animal on day − 2, − 1, 0, 1, 2, 7, and 14 in relation to the day of weaning (d 0). Observations were performed directly in the field using a recording sheet containing animal number, date, and time (at 3-min intervals) whereas the behaviour performed by the animals was recorded by two observers during the periods: 7 A.M. to 9 A.M., 11 A.M. to 1 P.M., 2 P.M. to 4 P.M., and 5 P.M. to 7 P.M. The behaviours recorded included grazing, ruminating, resting, suckling, walking, vocalising (mooing), and drinking as described^18,19^.

### Cortisol and non-esterified fatty acids (NEFA)

Concentrations of cortisol in plasma were determined in a single assay according to the manufacturer’s instructions (ADVIA Centaur^®^ Cortisol assay; Siemens Healthcare Pty Ltd, Bayswater, Victoria, Australia). Assay sensitivity was 2.0 ng/mL, and the intra-assay coefficient of variation was 3.5%. The NEFA were also determined in a single assay (Randox Australia Pty Ltd, Parramatta, Australia) previously used in cattle^20^. Assay sensitivity was 0.03 mEq/L, and the intra-assay coefficient of variation was 1.4%.

### Sample preparation for metabolome profiling

Sample preparation and acquisition methods for ^1^H NMR were based on published protocols^21,22^. Plasma was allowed to thaw a room temperature and an aliquot (350 µL) was mixed with 350 µL of aqueous (80% H_2_O:20% D_2_O) phosphate buffer solution including 0.075 M NaH_2_PO_4_, pH = 7.4 (KOH adjusted), 0.1% sodium azide, 1 mM 3-trimethylsilyl-1-[2,2,3,3,-^2^H_4_] propionate (TSP) in Eppendorf tubes. Samples were placed on a vortex for 30 s and then centrifuged at 6000×*g* for 10 min. Aliquots of the supernatant (600 µL) were transferred into 5 mm NMR tubes for ^1^H NMR analysis.

### Acquisition and analysis of ^1^H NMR

^1^H NMR spectra were acquired with a Bruker Avance III 400 MHz spectrometer operating at 400.13 MHz for ^1^H at 310 K equipped with a 5 mm broad-band inverse configuration probe. Samples were analysed in random order and automation with a SampleCase 24 sample automation system. Samples were analysed using water suppressed 1D NMR spectrum using the NOESYPRESAT pulse sequence (160 transients) and a Carr-Purcell-Meiboom-Gill (CPMG) spin echo sequence with pre-saturation (160 transients). Irradiation of the solvent (water) resonance was applied during pre-saturation delay (2.0 s) for all spectra and for the water suppressed 1D NMR spectra also during the mixing time (0.1 s). The pulse sequence parameters including the 90° pulse (~ 12 µs), pulse frequency offset (~ 1880 Hz), receiver gain (90.5), and pulse powers were optimised on a representative sample and then set constant for the cohort analysed. The spectral width was 30 ppm for NOESY experiments and 20 ppm for CPMG experiments. The resulting Fourier induction decays were processed with an exponential line broadening of 0.3 Hz prior to Fourier transformation, which were collected with approximately 32 k real data points.

^1^H NMR raw datasets were automatically phased, baseline corrected and referenced to the α-C_1_H glucose doublet (5.23 ppm) using MATLAB 7.0 software (MathWorks, Natick, MA). To reduce analytical variation between samples, the residual water signal (4.67–4.98 ppm) was truncated from the data set. Probabilistic quotient normalisation was performed across the cohort^23^. Assignment of endogenous metabolites was made to a high confidence using Chenomx^®^ (Chenomx Inc., Edmonton, AB, Canada) and by reference to published literature, online resources, and spiking experiments^24–26^. Statistical Recoupling of Variables^27^ was used to cluster pre-processed NMR spectrum data into features or peaks representing the relative metabolite abundance.

### Statistical analyses

Following the processing of the ^1^H NMR data, multivariate statistical analysis was performed using both MATLAB 7.0 and SAS (SAS Inc, Cary, NC). Data for behaviour, ADG, cortisol, NEFA and the relative abundance of identified metabolites were analysed using a mixed-effects linear regression model with treatment as a fixed effect, time as a repeated measure subjected to the random effect of calf, and treatment × time interaction. Sex was originally included in the model with the respective interaction, but this proved to be insignificant (P > 0.05) and thus deleted from the model. The spatial power covariance structure was selected based on the lowest Bayesian Information Criterion, which accounts for the uneven distance between repeated measures. All variables and residuals were tested for normality, random distribution and mean of zero. Data for cortisol and NEFA were transformed to log_10_ before analysis to normalise the distribution. The behavioural data was used to calculate the percentage of observations of each behaviour for each animal and day, and then data transformed using the arcsine square root before statistical analysis. Differences between treatment means were calculated within each point in time using pairwise comparisons.

Principal component analysis (PCA) on the normalised spectra was used to identify potential unusual spectra (outliers) and detect obvious patterns or trends in the metabolite profiles^28^. No outliers were detected and then a second PCA analysis was done with the relative abundance of the 26 identified metabolites at each time point to detect the clustering of treatments groups after plotting principal components. Orthogonal partial least-squares discriminant analysis (OPLS-DA) was used to discriminate animals between the treatment groups (supervised classification). These OPLS-DA models identify spectrums or abundance of metabolites that allow assigning each animal to a particular treatment group. The OPLS-DA models generated were run through 1000 cross-validation permutation testing to assess the validity of the supervised models^28–31^. The OPLS-DA models were generated from both the entire ^1^H NMR normalised spectra and the 26 identified metabolites to determine if a dataset was better at discriminating animals between weaning groups. The predictive ability of OPLS-DA models was measured by the Q^2^ value^30^.

## Results

### Pasture availability, ADG, and behaviour

**Figure 1:**
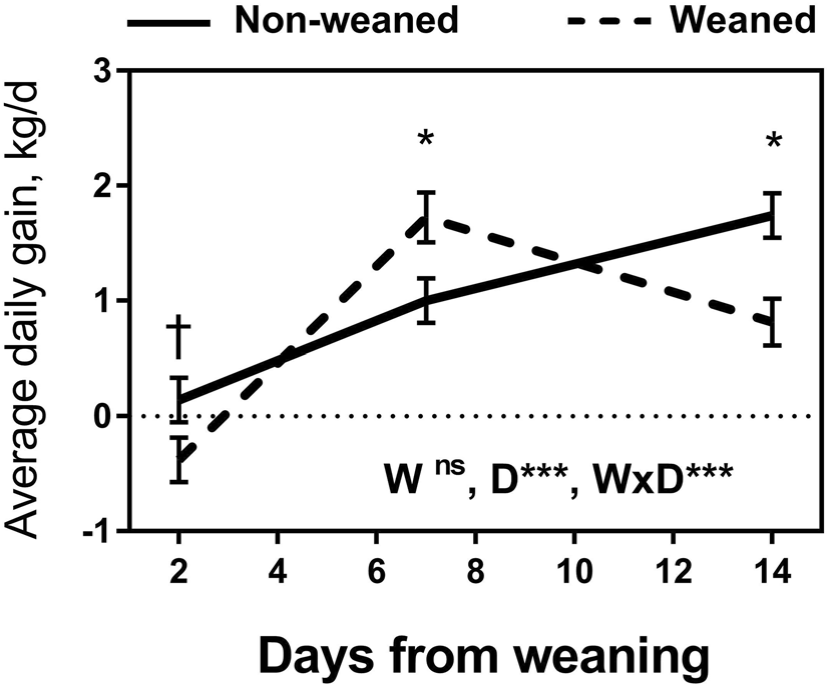
Growth rate of weaned and not weaned Angus calves. Weaning was at day 0. W is main effect of weaning; D is main effect of Day relative to weaning. †, *, **, *** values within day differ or tended to differ between treatment groups (P ≤ 0.10, P ≤ 0.05, P ≤ 0.01, P ≤ 0.001).

**Figure 2:**
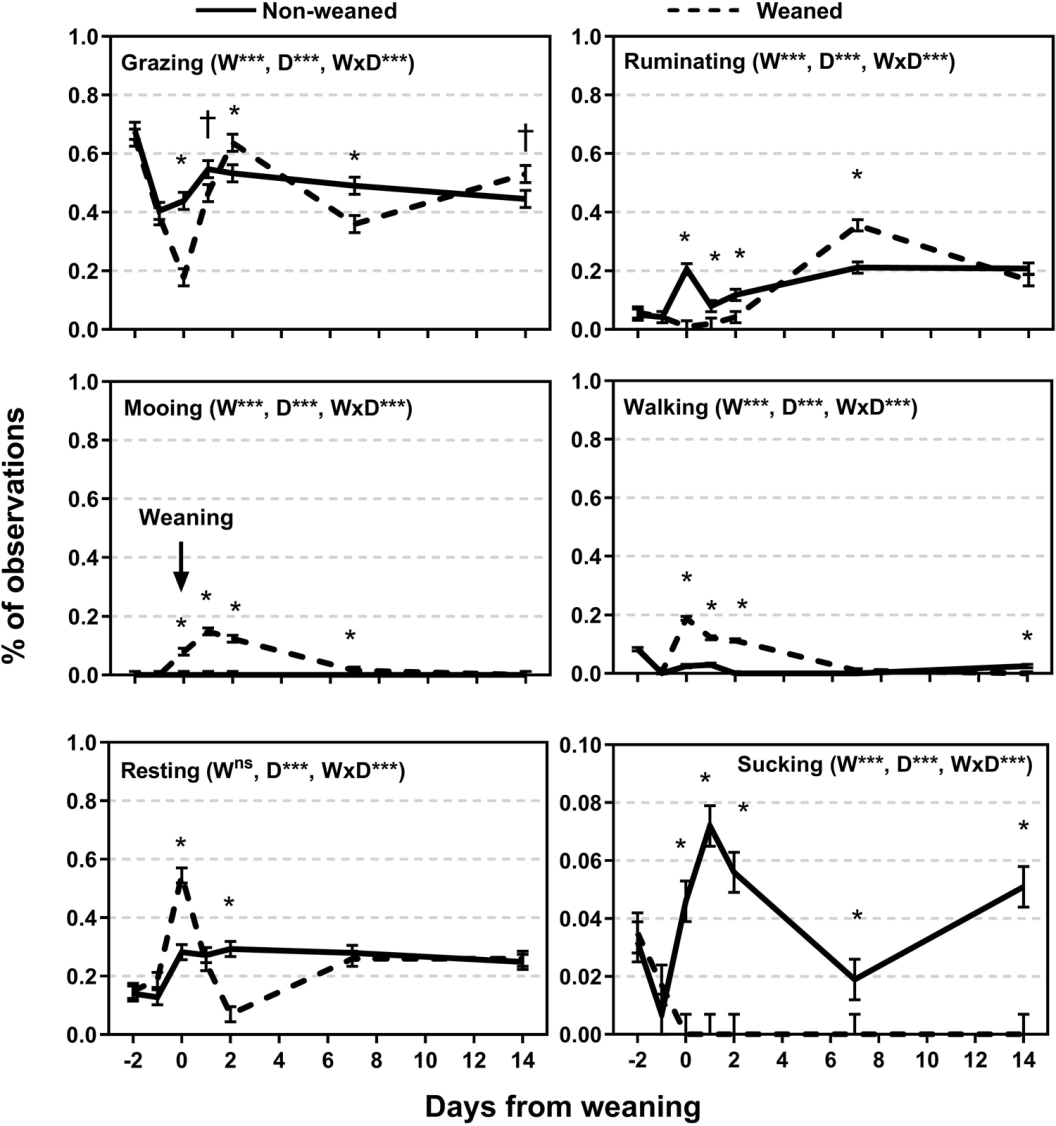
Behaviour of weaned and not weaned Angus beef calves relative to the time of weaning (day 0 was after weaning). W is main effect of weaning; D is main effect of Day relative to weaning. †, *, **, *** values within day differ between treatment groups (P ≤ 0.10, P ≤ 0.05, P ≤ 0.01, P ≤ 0.001).

No significant differences (P > 0.05; data not shown) were found between treatments in green or dry pasture biomass at the start (2130 ± 217 and 555 ± 67 kg DM, respectively) or end (1753 ± 105 and 668 ± 47 kg DM, respectively) of the trial. The BW on d 0 and d 14 did not differ (P > 0.05) between treatments (data not shown). Overall ADG was greater (P = 0.014) for NW calves (1.05 ± 0.079 kg/d) compared with W calves (0.74 ± 0.084 kg/d). The treatment × day interaction (P < 0.001) indicated that W calves lost weight between d 0 and d 2 while NW calves gained weight in this period (P = 0.06; Fig. 1). The ADG between d 2 and d 7 was greater in W compared to NW calves (P < 0.05) and the opposite effect was found between d 7 and d 14 (P < 0.05; Fig. 1). All behaviours showed a treatment × day interaction (P < 0.001; Fig. 2). Both W and NW calves spent a similar amount of time for each activity on d − 2 and − 1 (P > 0.10). In contrast, W calves spent a shorter time grazing compared to NW calves on d 0 (P < 0.05), d 1 (P = 0.06) and d 7 (P < 0.05) but more on d 2 (P < 0.05). W calves spent less time ruminating and more time vocalising, and walking compared to NW calves on d 0, d 1 and d 2 (P < 0.05; Fig. 2). There were no behavioural differences (P > 0.05) between W and NW calves at d 14 except for a shorter time spent sucking from the dams and longer walking in W compared to NW calves, respectively (P < 0.05).

### Blood physiology

**Figure 3:**
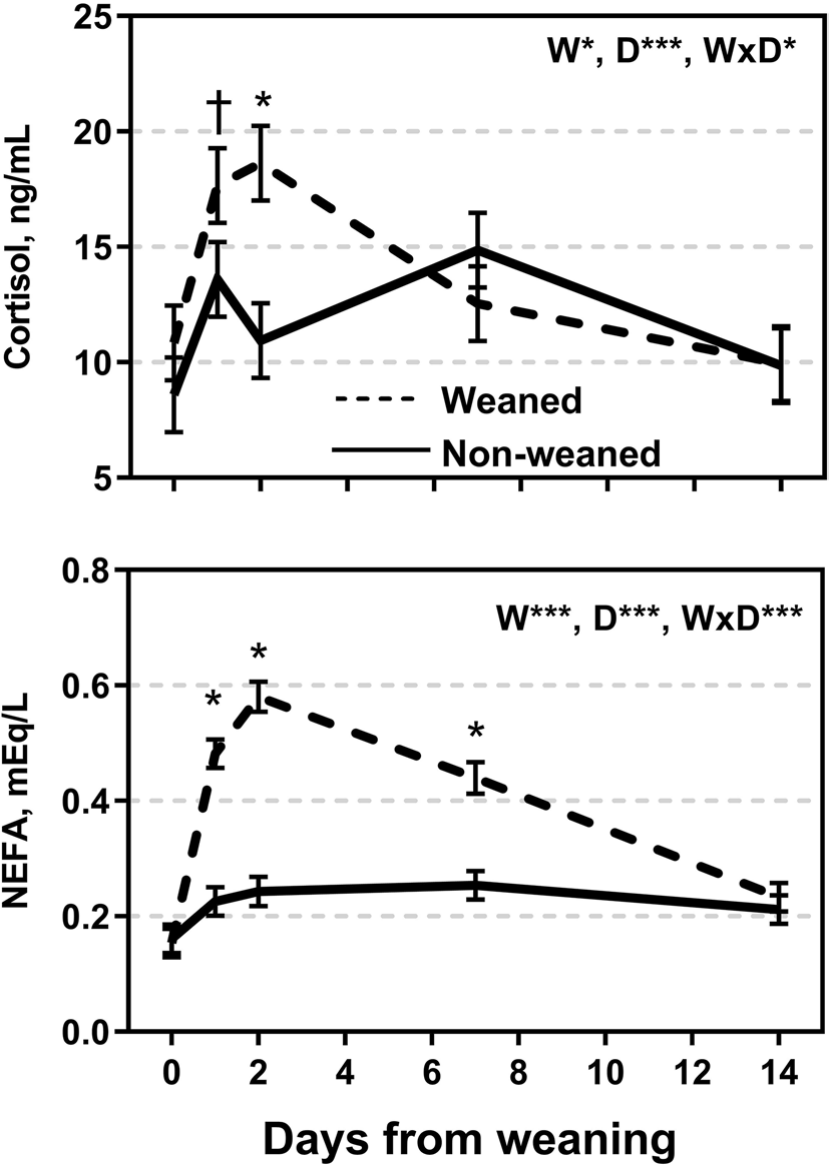
Blood concentration of cortisol and non-esterified fatty acids (NEFA) for weaned and not weaned Angus beef calves relative to the time of weaning at day 0 (blood sample before weaning). W is main effect of weaning; D is main effect of Day relative to weaning. †, *, **, *** values within day differ between treatment groups (P ≤ 0.10, P ≤ 0.05, P ≤ 0.01, P ≤ 0.001).

A weaning × day interaction was observed for plasma concentrations of cortisol (P < 0.05) and NEFA (P < 0.001; Fig. 3). The W calves tended to have greater concentration of cortisol at d 1 (P < 0.10) and 2 (P < 0.01) compared to NW calves but no differences between groups were observed at d 7 and d 14 (P > 0.10). Plasma concentrations of NEFA were greater (P < 0.01) in W compared to NW calves on d 1, d 2 and d 7 (P < 0.05), with no differences at d 0 and d 14 (P > 0.10; Fig. 3).

### Blood Metabolome

**Figure 4:**
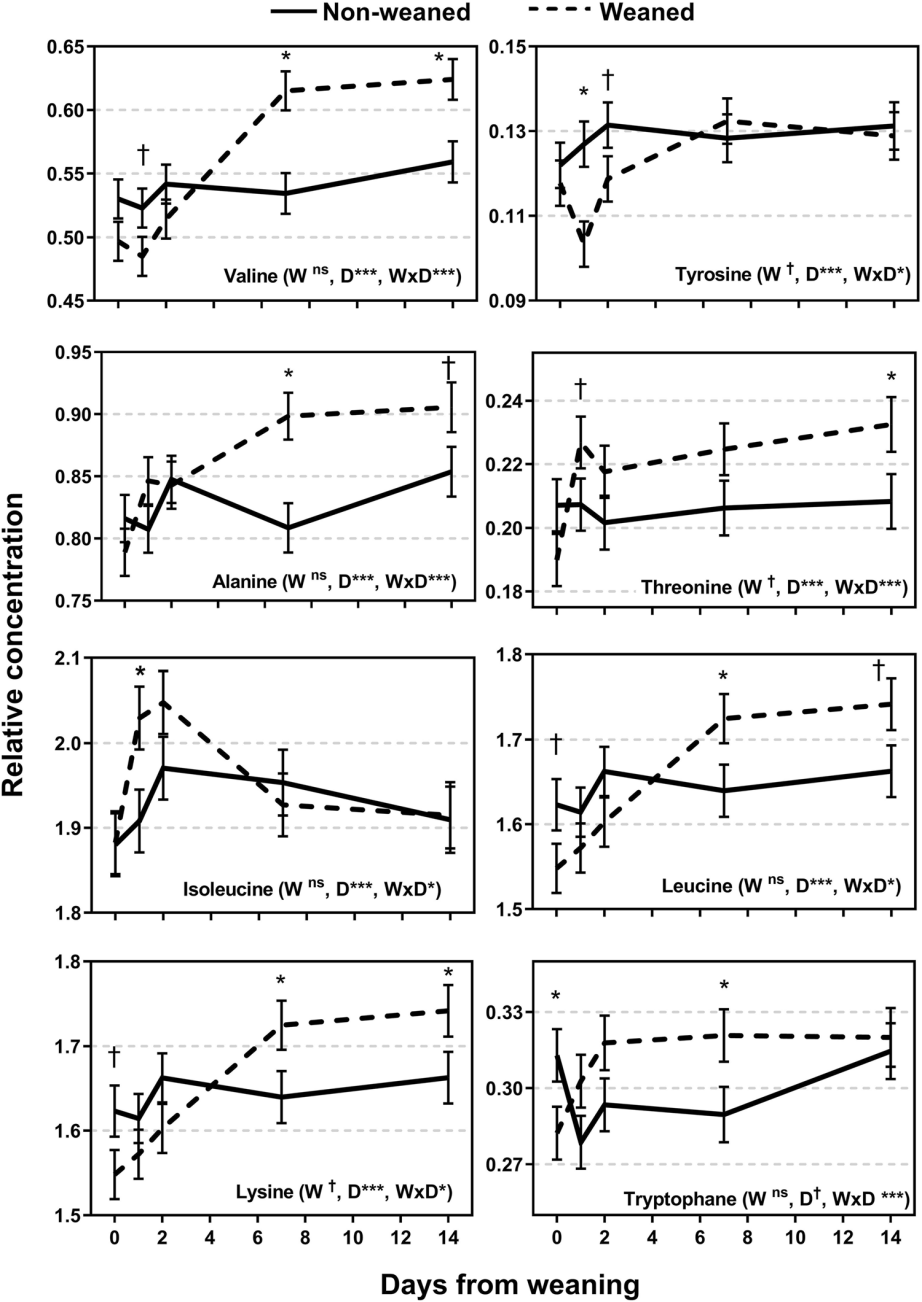
Relative concentration of plasma metabolites of weaned and not weaned Angus beef calves relative to the time of weaning at day 0 (blood sample before weaning). W is main effect of weaning; D is main effect of Day relative to weaning. †, *, **, *** values within day differ between treatment groups (P ≤ 0.10, P ≤ 0.05, P ≤ 0.01, P ≤ 0.001).

**Figure 5:**
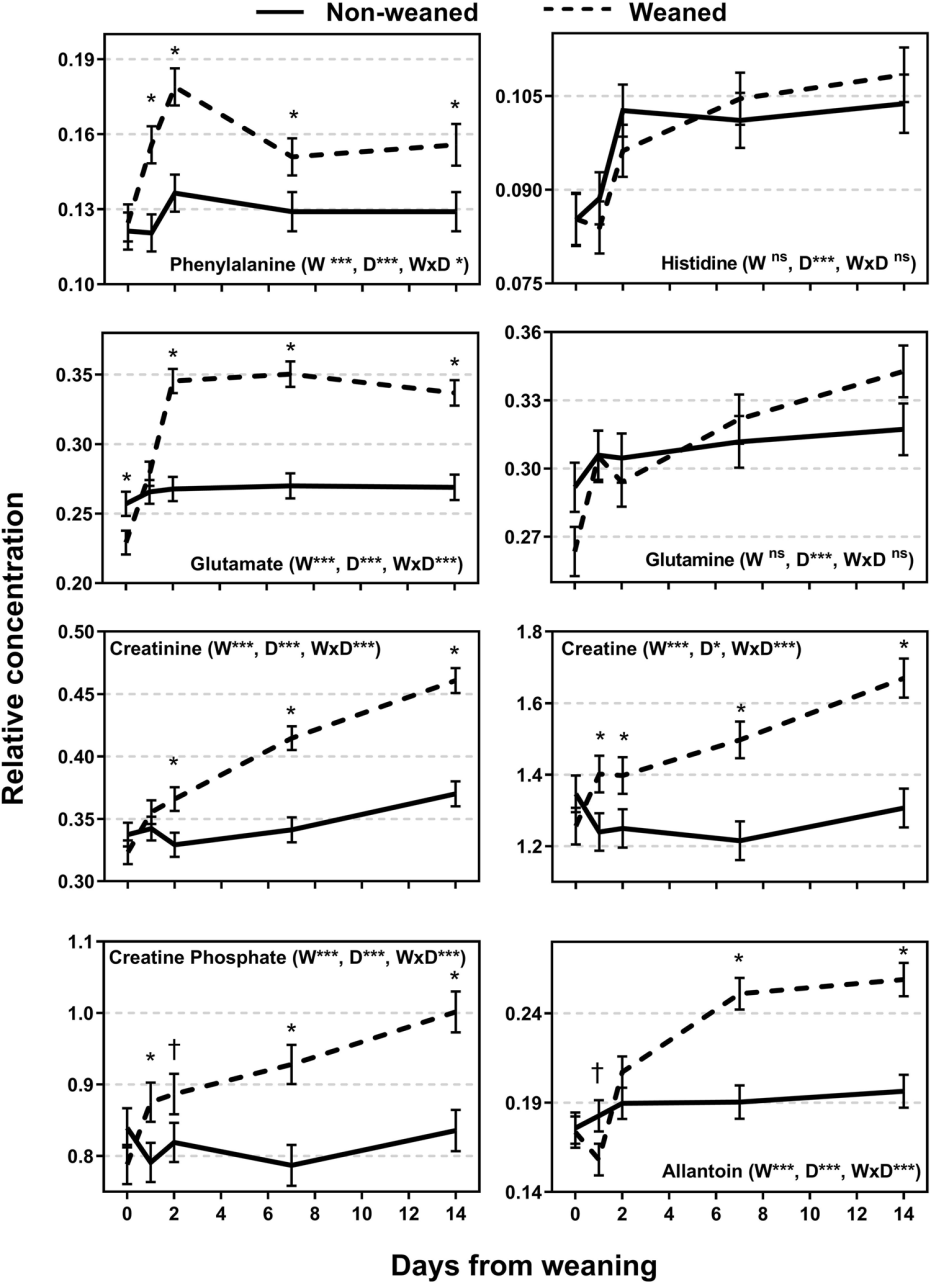
Relative concentration of plasma metabolites of weaned and not weaned Angus beef calves relative to the time of weaning at day 0 (blood sample before weaning). W is main effect of weaning; D is main effect of Day relative to weaning. †, *, **, *** values within day differ between treatment groups (P ≤ 0.10, P ≤ 0.05, P ≤ 0.01, P ≤ 0.001).

**Figure 6:**
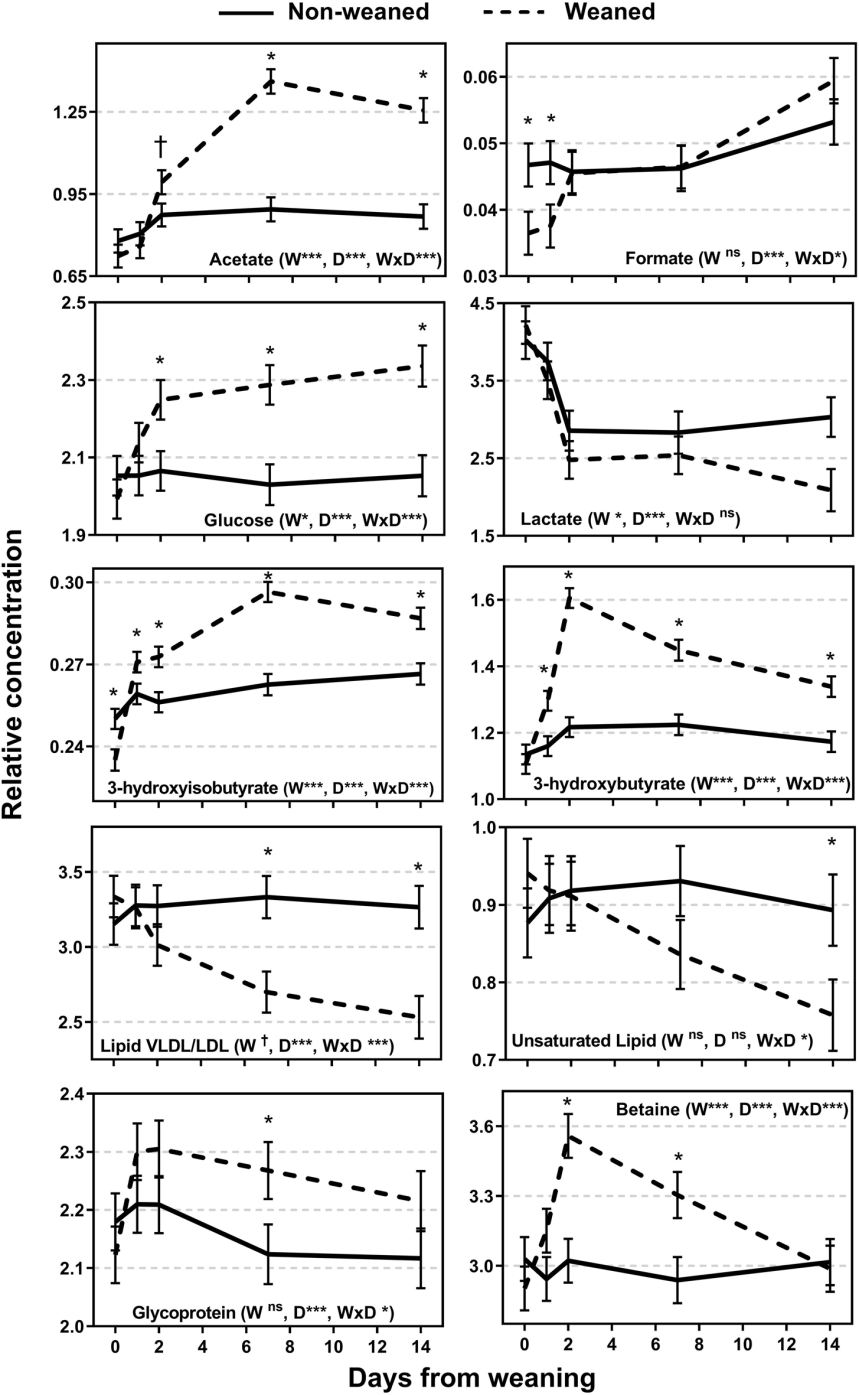
Relative concentration of plasma metabolites of weaned and not weaned Angus beef calves relative to the time of weaning at day 0 (blood sample before weaning). W is main effect of weaning; D is main effect of Day relative to weaning. †, *, **, *** values within day differ between treatment groups (P ≤ 0.10, P ≤ 0.05, P ≤ 0.01, P ≤ 0.001).

A total of 26 metabolites were identified from the ^1^H NMR spectra (Figs. 4, 5, and 6). The relative concentration of metabolites for NW calves did not change over time (P > 0.05) whereas all metabolites changed over time for W calves (P < 0.05). Differences between NW and W calves emerged after weaning for 24 of the 26 metabolites. The exceptions were the AA histidine and glutamine which did not show a treatment × day interaction (P > 0.05). Some metabolites showed the greatest difference between weaning groups at d 1 and d 2 and others at d 14 (Figs. 4, 5, and 6). Weaned calves had lesser abundance of tyrosine and greater abundance of isoleucine compared to NW calves on d 1 or d 2 (P < 0.10) with no differences at d 0, d 7 and d 14 (P > 0.10). Betaine was greater in W compared to NW calves on d 2 and d 7 (P < 0.05; Fig. 6). The relative abundance of other metabolites was greater for W compared to NW calves only at d 7 or d 14, or both d 7 and d 14 including a group of AA (valine, alanine, threonine, leucine, lysine, threonine), allantoin, acetate, and glycoprotein; and lower abundance in W compared to NW calves for VLDL/LDL and unsaturated lipids (P < 0.05). Another group of metabolites showed greater relative abundance in W compared to NW calves from d 1 or d 2 to d 14 including phenylalanine, glutamate, creatinine, creatine, creatine phosphate, glucose, 3-hydroxybutyrate, and 3-hydroxyisobutyrate (P < 0.10; Figs. 4, 5, 6).

**Figure 7:**
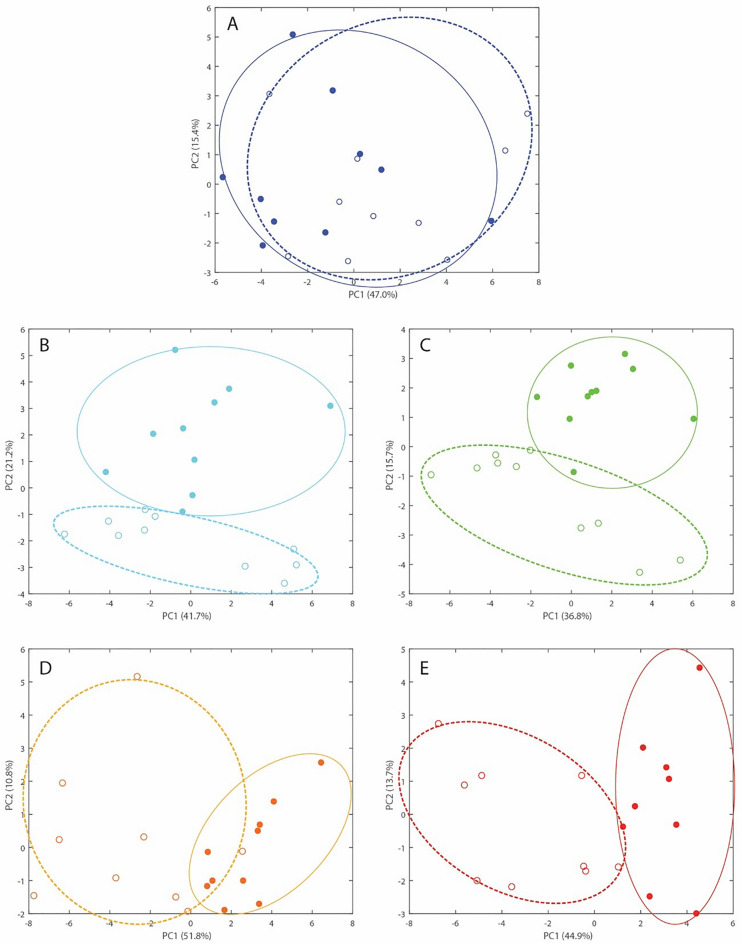
Principal Component Analysis of the ^1^H NMR spectra of non-weaned (open circles) and weaned (closed circles) Angus calves (**A**) immediately before abrupt weaning, (**B**) d 1, (**C**) d 2, (**D**) d 7, and (**E**) d 14 after weaning. The ellipses serve to highlight the lack of clustering at d 0 and the clustering of the non-weaned (dotted outline) and the weaned group (solid outline) on different days after weaning.

The unsupervised PCA plots with the 26 identified metabolites as predictors in Fig. 7 represent the first principal component (PC1, X-axis) which accounts for as much of the variability in the data as possible plotted against the second principal component (PC2, Y-axis). The PC2 accounts for the greatest possible variance in the data under the constraint that it is orthogonal to the preceding component. The variance explained by the first two PCs was over 50% for any day in relation to weaning (Fig. 7). Before weaning (d 0), the PCA did not display an obvious discrimination between NW and W calves based on their metabolomes as shown by the overlap of the two groups of calves (Fig. 7A). At d 1 after weaning, the PCA showed a separation between NW and W calves (Fig. 7B). This separation remained evident at d 2 (Fig. 7C), d 7 (Fig. 7D) and d 14 (Fig. 7E).

**Figure 8:**
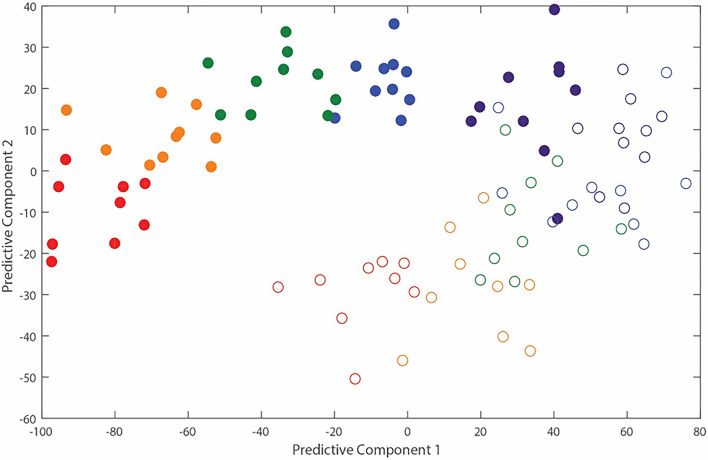
Stability of the metabotypes of Angus calves related to time and weaning treatment group (weaned or non-weaned calves). Two dimensional OPLS model scores plot (2 predictive and 5 orthogonal components) derived from ^1^H NMR spectra from the plasma of Angus calves (time point, *dark blue through to red*) that were non-weaned (*open circles*) and weaned (*closed circles*). Time points are d 0 (purple), 1 (blue), 2 (green), 7 (orange), and 14 (red) after weaning.

A multi-vector supervised OPLS-DA scatter plot using the entire spectrum of each sample also highlighted the differences in the blood metabolome of calves depending on time and weaning status (Fig. 8). The blood metabolome was markedly altered at d 1 after weaning which continued to d 14 after weaning. The increasing separation between W and NW calves with time indicated that the metabolome continued to diverge with increasing time after weaning.

Separate discriminatory models were subsequently created for each day of blood collection and either using the entire ^1^H NMR spectrum or the 26 identified metabolites (Table 1). At d 0, the Q^2^ value indicated that the OPLS-DA had no predictive validity of whether calves belonged to the NW or W groups (Table 1). The Q^2^ values for d 1 to 14 were > 0.40 which confirmed that the OPLS-DA could accurately discriminate between NW and W calves using both the entire spectrum and the relative abundance of metabolites (Table 1). Predictions using the metabolites seemed to perform slightly better than the entire spectrum due the slightly higher Q^2^. Random permutation testing confirmed all models were able to differentiate between treatment groups after the change in weaning status.

**Table 1:** Calculated Q^2^ values for each OPLS-DA model to predict weaning status in Angus calves weaned immediately after blood sampling on d 0 using either the entire ^1^H NMR spectrum or the relative concentration of metabolites.

Time after weaning, d	Q^2^ value from whole NMR spectrum	Q^2^ value from the 26 identified metabolites only
0	0.02—not valid	0.05—not valid
1	0.52—valid	0.64—valid
2	0.43—valid	0.58—valid
7	0.50—valid	0.60—valid
14	0.62—valid	0.70—valid

## Discussion

The present study sought to characterize the effect of abrupt weaning on the blood metabolome of beef calves measured using ^1^H NMR and supported by traditional measurements of stress and energy balance such as ADG, cortisol, NEFA, and behaviour. The PCA at each sampling date was performed to assess potential clustering of calves into either a W or NW group according to the metabolome as an unsupervised classification method. This analysis confirmed that weaning had major effects on metabolic function to allow the separation of groups based purely on the blood metabolome. These results were further confirmed using supervised classification (OPLS-DA), which showed that accurate discrimination can be achieved either using the entire NMR spectrum or the identified metabolites. Furthermore, both PCA and OPLS-DA analysis demonstrated that the metabolome of W and NW calves did not differ before weaning but a clear divergence of groups existed since the time of weaning. This is the first published study to assess the changes in the blood metabolome of beef calves after weaning to the authors’ best knowledge. Differences in the blood metabolome between NW and W calves were not explained by pasture total or green biomass available, which was similar for the two groups. Therefore, the metabolic changes observed in W calves are likely due to a combination of physiological and psychological stressors, type of nutrients consumed, and changes in metabolic function as a result of the discontinuation of milk intake after physical separation of the calf from the dam, cessation of suckling, removal of social support from the dam, and change of social organisation^2,4,32^. It is important to note that calves of the present study were 5 to 6 months-old and the development of the reticulorumen is expected to be completed with solid feed available at this age^33^. However, calves still suckled during the present study suggesting that a proportion of the energy and nutrients consumed came from the dam’s milk. Milk consumed bypasses the rumen to be digested in the abomasum and absorbed in the lower gastrointestinal tract^33^. The reduction in plasma relative concentration of VLDL/LDL and unsaturated lipids in W calves from d 0 to d 14 may reflect the abrupt reduction in fat intake from milk and reliance on forage intake. Forages have very low fat content (~ 1% of DM) whereas bovine milk has high fat content of approximately 30% of DM^33^.

In contrast to the long-term decline of lipids, the marked differences between W and NW calves during the first week after weaning in the present study could reflect physiological changes from stress hormones. Prior research concluded that the stress of weaning was acute and of short duration (up to 48 h) if the conditions are appropriate^2^. Findings of the present study for ADG, cortisol, and behaviour (vocalising, walking and grazing) support this observation because these variables returned to pre-weaning values by d 7 in agreement with previous studies^19^. Elevated blood cortisol influences a broad range of physiological processes in cattle including metabolism^34,35^ and immune function^5,6,36^. In the present study, the concentration of NEFA, betaine and 3-hydroxybutyrate also peaked at d 2 after weaning but remained elevated until d 7 and 14, respectively. In contrast, cortisol and behaviours indicative of stress had already returned to pre-weaning values by d 7. Elevated NEFA, betaine, and 3-hydroxybutyrate in cattle generally reflect an increase in lipolysis and body fat mobilisation which can be caused by stress-mediated glucocorticoids, high energy demand, or negative energy balance^2,37^. The W calves lost weight at d 2 after weaning however both NW and W calves gained weight thereafter indicating there was no negative energy balance. Therefore, the finding that W calves had lower ADG compared to NW calves suggests a cortisol-mediated effect on energy utilisation or a nutritional challenge from lower nutrient intake, or both.

The temporal trends in the abundance of NEFA for W calves differed markedly from those of lipids suggesting that these compounds do not share the same metabolic pathway despite both being part of lipid metabolism. This speculation is supported by a lack of effect of increasing fat concentration in milk replacer on blood NEFA and triglycerides of calves^38^. Furthermore, there was no correlation between NEFA and lipids in the present study (P > 0.05; data not shown). Therefore, lipids measured by ^1^H NMR may derive from dietary sources whereas NEFA may be derived from fat mobilisation.

Betaine is a methyl donor that participates in protein and lipid metabolism, improves the antioxidant capacity to protect cells from stress, and has osmoregulatory properties in cells^39^. In transition dairy cows, the production of betaine increased under negative energy balance, leading to an increase in plasma concentration of NEFA and 3-hydroxybutyrate to support energy production via lipolysis when plasma glucose concentration decreased^40^. Furthermore, betaine supplementation during periods of heat stress improved milk production in dairy cattle^41,42^ and growth and fat deposition in beef cattle^43,44^. It is considered likely that W calves of the present study increased betaine production to improve lipolysis and oxidation of triglycerides and thus, supply energy required for the stress response of weaning. However, the present findings differ from lactating dairy cows because calves were not in negative energy balance and glucose concentration increased in W compared to NW calves, unlike transition dairy cows.

The relative abundance of tyrosine decreased in W calves in the first 2 days after weaning in the present study, suggesting tyrosine also played an important role in the stress response to weaning. Tyrosine is a precursor of several neurotransmitters of the catecholamine pathway involved in the stress response including dopamine, norepinephrine and epinephrine^45–47^. It is hypothesized that the acute stress response to weaning increased the demand for and utilisation of tyrosine leading to a reduction of its concentration in blood. However, research on the role of tyrosine in the stress response of cattle is scarce and more research is required to draw more informed conclusions.

Glycoproteins are acute phase proteins that increase with inflammation, infection, stress, and trauma^48^. The increase in the relative abundance of glycoprotein in W calves compared to NW calves may have reflected the general stress response of W calves to abrupt weaning. However, acute phase proteins are generally not specific, and it is unclear if glycoproteins measured by NMR are the same as those measured by other techniques such as ELISA or chromatography. Nevertheless, these findings are important because demonstrate the potential of blood metabolomics to measure multiple metabolites that indicate different responses and metabolic pathways.

In contrast to the metabolites that seemed to reflect the acute stress of weaning, other metabolites reached their greatest relative concentration at d 14 such as valine, alanine, leucine, lysine, creatinine, creatine, creatine phosphate, acetate, and glucose. It is suggested that these metabolites reflect a change in metabolic function either due to diet change, chronic stress, or both. The fact that no significant changes in the metabolome of NW calves occurred over time in the present study suggest that these metabolites reflect metabolic adjustments of W calves. However, the present study only measured the metabolome to 14 d after weaning and it would be of interest to measure metabolites for a longer period after weaning to determine if these changes are a result of long-term metabolic changes with the cessation of milk intake or, alternatively, short-term metabolic adjustments or a consequence of the acute stress response.

The sharp and sustained increase in circulating glucose of W compared to NW calves could be interpreted to reflect an increase of gluconeogenesis either because of the stress response or metabolic adjustments to produce more energy from ruminal volatile fatty acids and gluconeogenic AA rather than from milk, or both. On one hand, glucocorticoids promote glucogenic metabolism during stress through the mobilisation of glycogen stores in liver and muscle^45,49–51^. However, cortisol and stress behaviours peaked at d 2 after weaning and returned to pre-weaning values by d 7 whereas glucose peaked at d 14 in the present study. On the other hand, the rate of gluconeogenesis increases with the transition from the pre-ruminant to the ruminant stage^1^. All calves of the present study consumed medium quality pasture and therefore dietary glucose was expected to be minimal. Gluconeogenesis in the liver may be supported by propionate absorbed from the rumen, lactose, lactate, and glucogenic AA such as alanine and glutamine^1^, and valine, isoleucine, and threonine^47^. Alanine, valine and threonine appeared to have supported gluconeogenesis in the present study increasing to d 14 like glucose. Propionate was not identified in the NMR spectra although acetate showed a large increase at d 7 and 14. However, lactate, glutamine, and isoleucine showed no differences between W and NW calves at d 7 or d 14.

Acetate is a VFA produced in higher proportion during fermentation of high forage diets in the rumen where it is absorbed into the bloodstream^52^. Therefore, the greater abundance of acetate in W compared to NW calves at d 7 and d 14 could have been due to higher intake of forage and fibre in W calves (partly substituting milk). After absorption, acetate can be converted to 3-hydroxybutyrate, oxidized via the tricarboxylic acid cycle (TCA) or used for fatty acid synthesis^53^. However, the concentration of acetate did not peak at the same time as 3-hydroxybutyrate in the present study.

The temporal trend of 3-hydroxybutyrate of W calves in the present study was interesting because it peaked at d 2 but remained elevated until d 14. This metabolite can originate from either incomplete oxidation of fatty acids during lipolysis or the conversion of acetate during absorption from the rumen^54^. Therefore, it is possible that the trends observed in W calves reflected both metabolic processes, with greater abundance of 3-hydroxybutyrate during the first 2 days after weaning reflecting incomplete hepatic oxidation of fatty acids (NEFA) mobilised during the acute stress response. The relative abundance of 3-hydroxybutyrate could has then been sustained by the conversion of acetate absorbed in the rumen due to increased forage intake. This is supported by results from previous studies which demonstrated that 3-hydroxybutyrate increased during periods of high energy demand or negative energy balance in post-partum dairy cows or calves after transport and weaning^37,55,56^. It is important to note that the relative contribution of different metabolic pathways to the observed trends and abundance of circulating metabolites cannot be measured with the experimental design of the present study. It is also possible that other metabolites also reflect the interaction between multiple metabolic pathways such as glucose, creatine, phenylalanine, glutamate, and 3-hydroxyisobutyrate.

Creatine, creatine phosphate and creatinine of W calves were maximally different to NW calves at d 14. These metabolites are critical in energy metabolism of the ruminant animal to store and recycle energy. Elevated creatine kinase, creatine and creatinine are associated with excessive muscle breakdown, muscle damage during periods of increased or unaccustomed physical activity and stress^45–47^. Creatine is one of the main energy sources for brain tissue and skeletal muscle during physical activity, which is converted to creatine phosphate by the enzyme creatine kinase and stored as a rich energy source^47^. This reaction is reversible and thus creatine phosphate can be converted to creatine to produce energy (ATP), and creatine can then be converted to creatinine in an irreversible reaction which is an inactive end product of muscle metabolism excreted in urine^47,57^. For this reason, creatinine is often used an indicator of proteolysis and body muscle mass, and typically decreases in blood concurrent with increases of 3-hydroxybutyrate and NEFA and with a reduction of glucose in post-partum dairy cows losing BW under negative energy balance^58,59^. However, these changes observed in dairy cows are in contrast with the results in weaned calves of the present study where all three metabolites increased to d 14 after weaning. The W calves of the present study showed increased physical activity immediately post-weaning at d 1 and d 2 but declined at d 7 and d 14, in agreement with previous studies^9,19^. Therefore, the increased abundance of creatine and creatinine of W calves to d 14 appears not to be explained by physical activity, behavioural indicators of stress, cortisol, or NEFA. It could be possible that the production of creatine and storage of creatine phosphate continues after a period of acute stress or high physical activity leading to ‘chronic’ elevation of these metabolites. However, this latter hypothesis cannot be confirmed with data from the present study and further research is needed to determine whether such profound and long-term changes in energy and protein metabolism are a result of earlier acute stress resulting in chronic elevation of creatine and creatinine or to metabolic changes due to discontinuation of milk intake. Regardless, W calves seemed to have been in a catabolic situation until d 14 with both proteolysis and lipolysis occurring as reflected by high relative concentrations of creatine, creatinine, 3-hydroxybutyrate, glucose, and some AA.

Allantoin increased at d 7 and d 14 in W compared to NW calves in the present study. Allantoin is one of the purine derivatives which increases with the flow of microbial protein from the rumen and with OM intake^60^, and it is also involved in the urea cycle and excreted in urine^47^. In addition, allantoin has been suggested as a biomarker of chronic oxidative stress, illness and senescence in both cattle and humans^61,62^. Chronic stress was not evident in the present study, and it is hypothesized that the greater allantoin concentration of W compared to NW calves is a result of greater solid feed intake and ruminal microbial activity after discontinuation of milk intake. However, feed intake was not measured in the present study and thus further research is needed to confirm this hypothesis. Similarly, the reasons for the sustained increase in valine, alanine, threonine, leucine, lysine, phenylalanine and glutamate in W calves to d 14 are difficult to explain in the present study because total protein intake was not measured (from both milk and pasture). Faecal N concentration measured on the same days of blood sampling as an indicator of protein intake showed no differences between W and NW calves at any time point with an overall average of 1.12 ± 0.033 and 1.15 ± 0.032% of DM for W and NW calves, respectively (data not shown; P > 0.05). This could imply that total protein flow to the lower gastrointestinal tract was not different between W and NW calves.

Previous research that measured metabolites in blood were undertaken in calves fed milk replacers which did not experience the stress from separation of the dam as in the present study. In addition, milk allowance was gradually reduced in most previous studies with dairy^63^ and beef calves^64^ and these studies have been done in small pens where animals cannot fully express their stress behaviours such as walking long distances. For instance, discontinuation of feeding milk replacer showed no effects on glucose or NEFA in blood^64,65^, and increased glucose, acetate, propionate and butyrate, and a decrease in NEFA^63^. Interestingly, 3-hydroxybutyrate increased after weaning in all the above studies and there is agreement this metabolite comes from the conversion of acetate and butyrate in the ruminal epithelium as a result of greater solid feed intake fermented in the rumen of weaned calves^63^. A study with beef calves at foot on pasture that were placed in pens at weaning produced similar results to the present study, where both NEFA and 3-hydroxybutyrate increased after weaning although glucose and creatine kinase were not affected^3^ unlike the present study. It is important to note that the latter study did not have a control NW group like the present study. A review of the effects of weaning on calves concluded that circulating glucose produced inconsistent results across studies but the causes for these discrepancies were not identified^2^.

The present study confirmed that blood metabolomics is a strong platform to investigate and understand metabolic changes resulting from complex interactions that arise from stress and dietary changes. The combination of high throughput ^1^H NMR and advanced statistics is a powerful combination that generates a large amount of biological information. The field of metabolomics can make an important contribution to increasing the understanding of complex metabolic changes. This could facilitate the development of strategies that enhance the management, performance and welfare of production animals. For example, supplements for calves at weaning could be formulated with high concentration of precursors of the metabolites required for gluconeogenesis or the stress response such as tyrosine. Furthermore, the nutritional requirements of weaned calves could be managed using new technologies to deliver targeted amounts and types of supplements^66^. These approaches can also be integrated with strategies that reduce physical activity such as yard weaning, fenceline contact, or two-stage weaning which are known to lower the stress response and energy expenditure^8,9^.

In conclusion, several key metabolites showed dramatic changes in calves to d 14 after weaning reflecting marked changes in carbohydrate, lipid, and protein metabolism due to both stress and diet change. Metabolic changes due to stress seemed of short duration being most marked on d 2 after weaning, whereas longer-term metabolic changes were observed until d 14 after weaning. The drastic short-term changes in lipid metabolism of W calves were reflected through a sharp increase of NEFA and betaine as methyl donor to facilitate lipid oxidation. The metabolite 3-hydroxybutyrate seemed to be involved in both short- and long-term changes in lipid metabolism peaking at d 2 and remaining high until d 14 after weaning. Long-term changes in lipid metabolism were shown through lower concentration of LDL/VLDL and unsaturated lipids, which were most evident at d 14 after weaning and could be the result of diet change from discontinuation of milk intake. The most important metabolic change associated with carbohydrate metabolism was the significant increase in glucose concentration in plasma of W calves until d 14, which could have been due to an increase of gluconeogenesis. In addition, a sharp increase in acetate observed on d 7 and 14 could reflect greater fermentation of solid feed in the rumen which may also have contributed to a sustained high abundance of 3-hydroxybutyrate of W calves. Changes in protein metabolism were evidenced through an increase in several AA and allantoin up until d 14. Finally, profound long-term changes in energy metabolism of peripheral muscle and brain tissue were reflected through a large increase in the relative concentration of circulating creatine, creatine phosphate, and creatinine in W calves up to d 14. Further research is needed to ascertain whether the long-term metabolic changes of calves after weaning are a permanent shift in metabolism or a transient change because of acute or chronic stress, and whether metabolite concentrations return to pre-weaning values.

## Data Availability

Data is available upon reasonable request to the corresponding author.
